# The Potential Roles of Extracellular Vesicles in Cigarette Smoke-Associated Diseases

**DOI:** 10.1155/2018/4692081

**Published:** 2018-11-06

**Authors:** A-Reum Ryu, Do Hyun Kim, Eunjoo Kim, Mi Young Lee

**Affiliations:** ^1^Department of Medical Science, Soonchunhyang University, 22 Soonchunhyang-ro, Asan, Chungnam, Republic of Korea; ^2^Department of Biology, The College of Wooster, 1189 Beall Ave, Wooster, OH, USA; ^3^Companion Diagnostics and Medical Technology Research Group, Daegu Gyeongbuk Institute of Science and Technology (DGIST), Daegu, Republic of Korea; ^4^Department of Medical Biotechnology, Soonchunhyang University, 22 Soonchunhyang-ro, Asan, Chungnam, Republic of Korea

## Abstract

Cigarette smoke contains more than 4,500 chemicals; most of which are highly reactive free radicals, which induce proinflammatory and carcinogenic reactions. Numerous efforts have focused extensively on the role of cigarette smoking as a cause of many diseases. Extracellular vesicles and exosomes have recently received increasing interest for their diagnostic and therapeutic roles in many diseases. However, research done on the role of extracellular vesicles and exosomes on cigarette smoke-induced chronic disease is still in its infancy. In this review, we summarize the recently addressed roles of extracellular vesicles and exosomes in the pathogenesis of cigarette smoke-related diseases, such as chronic obstructive pulmonary disease, cardiovascular disease, lung cancer, and oral cancer. Moreover, their potential utilization and future prospects as diagnostic biomarkers for cigarette smoke-related diseases are described.

## 1. Introduction

More than 6 million deaths are attributable to direct tobacco use per year globally, according to the World Health Organization (WHO) (2017). The death toll is expected to rise to 10 million per year by the 2020's or early 2030's. Cigarette smoking was the cause of 1 in 5 deaths in the United States and 1 in 3 deaths among men older than 30 years of age in Korea [1]. Cigarette smoke (CS) contains more than 4,500 chemicals; most of which are highly reactive free radicals, including peroxy radicals, nitrogen radicals, and other oxygen-derived species, which induce proinflammatory and carcinogenic reactions [2]. Thus, cigarette smoking causes serious health problems associated with oxidative and nitrosative stress and is the most relevant risk factor for chronic obstructive pulmonary disease (COPD), cardiovascular disease, and lung and oral cancers [3, 4].

Numerous efforts have focused extensively on the role of cigarette smoking as a cause of many diseases, studying the epidemiological and biomedical mechanisms. Moreover, despite the recent progress in research on extracellular vesicles (EVs) and exosomes as diagnostic and therapeutic targets in many diseases, the understanding of the role of EVs and exosomes associated with cigarette smoking is in its infancy. EVs are heterogeneous and include exosomes, microvesicles (microparticles or extosomes), and other vesicles still under controversy. Thus, we use the term “EV” in this review, except for instances where we clearly refer to exosomes.

EVs and exosomes are nanosized particles that participate in intercellular communication via delivery of crucial molecules to both distant and adjacent cells [5]. Exosomes are enriched with several molecular cargos, such as DNAs, mRNAs, miRNAs, and proteins, affecting diverse biological processes of the recipient cells [5–7]. Significantly enriched biomarkers via exosome shedding can be detected in certain disease states, whereas they are undetectable under normal conditions, suggesting that exosomes could play a role as diagnostic signatures reflecting the physiological condition of the parental cells [8, 9]. Cancer cell-derived exosomes might deliver miRNAs or proteins to recipient cells to trigger a favorable microenvironment for the proliferation and metastasis of tumors via horizontal exchange of tumorigenic information [9].

The research focusing on the role of EVs and exosomes in CS exposure-associated chronic diseases is limited; approximately 70 articles on this topic can be found in PubMed and Google Scholar. Exposure to cigarette smoke (CS)/extract (CSE) stimulated EVs and exosome release into the serum, saliva, urine of smokers/ex-smokers, and cancer patients, as well as from various cultured cells [10, 11]. Moreover, exosome secretion by CS led to several biochemical and cellular processes, including angiogenesis [12], endothelial dysfunction [8], and tissue remodeling [13], as well as proinflammatory [14] and prothrombotic effects [15, 16], promoting the pathogenesis of CS-related chronic diseases as categorized by Benedikter et al. [12, 17, 18].

Recently, a number of studies have suggested that oxidative stress affects the release of EVs in various cell types and human samples [15, 16, 19]. Although the mechanism underlying exosome release by cigarette smoke is not fully elucidated, redox-dependent thiol modification seems to be a plausible explanation for the exosome release under stress condition by CS [18]. The pathophysiological and cellular processes implicated in cigarette smoke-triggered exosome release are suggested to be the potential mechanisms that account for cigarette smoke-induced pathogenesis [6]. In this review, we briefly introduce and summarize the recently addressed roles of EVs and exosomes in the pathogenesis of cigarette smoke-related diseases, such as COPD, cardiovascular disease, lung cancer, and oral cancers. Moreover, the future prospects and potential utilization of EVs and exosomes as biomarkers for the diagnosis of cigarette smoke-related diseases are described.

## 2. The Release of EVs in Response to CS Exposure

EVs, induced under oxidative stress conditions, exhibit proinflammatory and prothrombotic effects which contribute to the pathogenesis of chronic diseases. The thiol-reactive compounds like acrolein, an endogenous inflammatory metabolite, contributed to exosome release by CS by reacting with cell surface thiols in the airway epithelial cells [18, 20, 21]. EV release was also enhanced with plasma membrane blebbing in response to thiol-reactive RCS (reactive carbonyl species) and ROS (reactive oxygen species). Moreover, EV shedding by thiol modifications protected the secreting cell against oxidative damage; however, at the same time, it induced a proinflammatory and prothrombotic state of the cells. However, both exosome release by CS [21] and the oxidation of protein thiols of the EV-secreting cells [18] were blocked by thiol antioxidants like N-acetyl cysteine (NAC) and glutathione S-transferase (GSH). These data clearly indicate that CS-induced EV release could be suppressed by thiol antioxidants. In other words, EV production under oxidative stress and a proinflammatory state was modulated by redox-dependent modification of protein thiols [12, 18]. In conclusion, thiol protection seems to be necessary for inhibiting the detrimental alterations caused by EV signaling under inflammatory and oxidative stress.

Recently, there is accumulating evidence that oxidative thiol modification promotes coagulation associated with EVs. The production of prothrombotic EVs was enhanced under oxidative and proinflammatory states with thiol depletion [22–24]. Phosphatidylserine (PS) [25] and tissue factor (TF) [13, 26] were implicated in the prothrombotic effect of EVs. Additionally, recent publications discuss in detail the targets of redox-dependent thiol modifications related with EV release, including exofacial thiols, protein disulfide isomerases, actin filaments, and redox-sensitive calcium channels. Taken together, thiols on the protein modulate EV release under thiol-depleting states via membrane fusion and blebbing, likely as an adaptation strategy against various oxidative stressors including CS.

## 3. Pathophysiological Processes Associated with CS-Induced Release of EVs

CS-triggered oxidative and nitrosative stress involved in the pathogenesis of several chronic diseases has been reported in diseases such as chronic obstructive pulmonary diseases (COPD), cardiovascular disease, lung cancer, and oral cancer. Biochemical and cellular processes, such as inflammation, prothrombosis, angiogenesis, endothelial dysfunction, and tissue remodeling, are ascribed to the pathogenesis of CS-related diseases [12]. Here, the recent literature, focusing on the most studied issues of inflammation and thrombophilia based on reported article numbers, is summarized in detail. They specifically imply a link between EV release by CS exposure and the biochemical processes at the cellular level.

### 3.1. Inflammation

The modulation of inflammatory responses associated with CS exposure-induced EVs was described in human peripheral blood mononuclear cells [25], human bronchial epithelial cells [17], macrophages [27, 28], and human epithelial cells [29], as well as in smokers with COPD [8] and lung cancer [14]. SOCS (suppressor of cytokine signaling proteins) is a gene family which regulates inflammation and Th cell differentiation via inhibiting the JAK-STAT signaling pathway. SOCS concentration in CS-exposed mice and BALF of human smokers were lower than those of the nonexposed groups [27]. Alveolar macrophages play a crucial role in host defense against respiratory tract infections via their phagocytic properties. In addition, they regulate host inflammatory response via cytokine production and anti-inflammatory microenvironment induction. Notably, the inflammatory response was suppressed by delivering alveolar macrophage-derived EVs with SOCS 1 and 3 to epithelial cells [27]. The internalization of alveolar macrophage-derived EVs by target cells was downregulated by CSE, showing loss of the EV-dependent anti-inflammatory state [30]. CSE-exposed mononuclear cells induced significant production of microparticles. They showed a proinflammatory potential with the expression of ICAM-1, IL-8, and MCP-1. In conclusion, CS inhibits EV-dependent anti-inflammatory signaling, whereas it activates EV-dependent proinflammatory signaling [28].

According to the results of Héliot et al. [14], the exposure of BEAS-2B cells to smoker EVs elevated IL-6 and IL-8 levels in comparison with exposure to nonsmoker EVs. The production of CYFRA21-1, a prognostic factor in non-small cell lung cancer (NSCLC) was also upregulated in BEAS-2B cells exposed to smoker EVs. In addition, a comparison of EV miRNAs between smokers and nonsmokers demonstrated that four miRNAs, miR-21, miR-27a, let-7g, and let-7e, which are potentially attributed to lung carcinogenesis and might be used as lung cancer biomarkers, were differentially expressed [14]. The expression of miR-21 and miR-27a in BEAS-2B cells increased after treatment with smoker EVs, when compared with exposure to nonsmoker EVs, whereas let-7e and let-7g expression was decreased with smoker EV treatment. The reduced expression of NF-E2-related factor 2 (NRF2), a key regulator of antioxidants implicated in lung carcinogenesis, was significantly and inversely correlated with the expression of its target miRNA, miR-27a.

Other indirect data on the effect of CS-exposed EVs in inflammation were also obtained from transcriptomic (microRNA) and proteomic analyses of EV contents via bioinformatics approaches. CS exposure to human samples resulted in a parallel increase in EVs and proinflammatory molecules, yet a clear association between EVs and inflammation has not been confirmed *in vivo*. Moreover, there is conflicting data regarding the ability of CS exposure to affect the release of EVs and inflammatory molecules in human blood [15]. The discrepancy might be due to the differences in EVs and exosome phenotypes, depending on age, gender, hormone profile, and smoking status [15, 19, 26]. The level and function of microvesicles differed with age; higher levels of microvesicles with CD41, CD235, tissue factor, and phosphatidylserine were found in the younger and older groups, compared to those of middle age [26]. Moreover, AREG on plasma EVs was decreased with increase in age, in both males and females [19]. With respect to cigarette smoking, the levels of microparticles with CD41 and CD45 were enhanced during active smoking of one cigarette, while CD144 did not change [15]. In the case of gender, plasma EVs from male smokers had higher levels of CD171, PD-L1, and TSG101 than those from female smokers. The levels of AREG, MUC1, CD146, CD13, and TSG101 in EVs were notably reduced in female smokers but not reduced in male smokers [19]. Thus, more in-depth research will help elucidate the mechanism by which CS induces exosome shedding and how the exosomes modulate inflammatory responses, contributing to chronic disease development and aggravation.

### 3.2. Thrombophilia

Thrombophilia (or hypercoagulability) is the abnormal tendency to develop blood clots. CSE-induced EV release is thought to be a significant cardiovascular risk factor for smokers. Tissue factor (TF) and phosphatidylserine (PS) in the EV membrane promoted thrombin production via assembly of coagulation factors [16, 26]. *In vitro* experiments showed increased release of endothelial EVs with PS and TF associated with procoagulant activity [8]. EVs derived from mononuclear cells in response to CSE express active TF, thus potentially contributing to the pathogenesis of cardiovascular diseases.

At present, information on the involvement of procoagulant EVs with TF and PS exposed to CSE *in vivo* is limited, probably due to small sample size, limitation of correction for confounding factors, and low sensitivity of antibody-based detection. In the case of PS, induction of EVs with PS expression [8, 16, 25] has been clearly detected *in vitro*. However, there are conflicting data on the levels of EVs with PS in the blood of smokers compared to nonsmokers [26, 31, 32]. Further research should be carried out to build on the current findings and understand whether EVs with PS could play a key role in thrombophilia caused by CS exposure *in vitro* and *in vivo*.

## 4. Chronic Diseases Associated with CS-Induced Release of EVs

### 4.1. Cardiovascular Disease (CVD)

Smoking is a major risk factor of cardiovascular disease (CVD), which is the leading cause of death worldwide. Smokers have a higher risk of developing vascular diseases such as atherosclerosis and venous thromboembolism than nonsmokers [33]. CS-induced alteration of lipid profile via oxidation by free radicals and oxidants originated from CS [34]. CS-induced CVD development occurs via several potential mechanisms such as inflammation, vascular dysfunction, thrombophilia, and atherosclerosis via lipid peroxidation, thrombus formation, and foam cell formation [35].

Microvesicles (MVs) released from human macrophages in response to CS possess proteolytic activity. The gelatinolytic and collagenolytic activities of CS-induced release of microvesicles from macrophages were predominantly ascribed to MMP14 production [13]. The MMP14-positive MVs may contribute to matrix damage, leading to instability of atherosclerotic plaques and emphysematous lung destruction [13]. CS exposure elevated the shedding of microparticles with proinflammatory and procoagulant potential in human mononuclear cells, via calcium-dependent mechanisms [25]. The roles of exosomes as biomarkers for acute coronary syndromes, myocardial infarction, and heart failure have been suggested. Cardiac-specific miRNAs, such as miRNA-1 and miRNA-133a, were released in injured cardiomyocytes and showed the highest plasma levels following the onset of myocardial infarction symptoms [36]. Moreover, circulating p53-responsive miRNAs, such as miRNA-192, miRNA-194, and miRNA-34a, have been reported to play as predictors of ischemic heart failure associated with acute myocardial infarction [37]. Circulating plasma microvesicles (PMVs) and their microRNAs are suspected to be major contributing factors for atherosclerosis, serving as biomarkers for CVD progression. However, little is known about how smoking affects PMV shedding and microRNA signatures *in vivo* [31]. Badrnya et al. provided evidence that smoking affects the PMV profile and microRNA cargo. Smokers showed increased levels of circulating leukocyte-derived PMVs (lPMV) and miR-29b and decreased levels of platelet-derived PMVs (pPMVs), the major PMV, and miR-223. They suggested that alteration of the PMV profile and miRNA, contributable to atherogenesis, could serve as an early biomarker in smoking-related diseases, despite sample sizes being small [31]. CS-exposed neutrophils accelerated the production of membrane microvesicles (MVs) with enzymatically active transmembrane ADAM proteases, ADAM10 and ADAM17. Production of ADAM10- and ADAM17-positive MVs from neutrophils on exposure to CS indicates the molecular mechanism underlying the dramatically elevated risk of abdominal aortic aneurysm (AAA) development in smokers [38].

### 4.2. Chronic Obstructive Pulmonary Disease (COPD)

COPD is a representative disease mainly triggered by inhalation of CS, despite the genetic aspect of COPD [39, 40]. The pathogenesis of COPD is manifested by airway inflammation (chronic bronchitis), degradation of lung tissue (emphysema), and progressive airway limitation associated with fibrotic airway remodeling. A small but significant pool of literature cites the importance of exosomes in CS-related COPD. Fujita et al. reported that the myofibroblast differentiation for airway remodeling in COPD pathogenesis was driven by exosomal miR-210 [10]. CS induced miR-210 expression in exosomes from human bronchial epithelial cells (HBECs). The upregulated miR-210 promoted myofibroblast differentiation in primary lung fibroblasts, whereas it suppressed autophagy via downregulating ATG7, an essential component of autophagy. Therefore, miR-210 was suggested to be a potential therapeutic target for COPD. In addition, downregulation of the exosomal let-7 (tumor suppressive miRNA) family following cigarette exposure was reported in the COPD model and lung cancer development.

The roles of exosomes in COPD can be inferred from the report by Moon et al. using endothelium-derived exosomes after exposure to CS [17]. CS upregulated full-length CCN1 expression in exosomes from epithelial cells, allowing transfer of inflammatory signals to distant portions of the lung. However, prolonged exposure to CS cleaved the full-length CCN1 in exosomes into the truncated form. The cleaved CCN1 activates the secretion of MMP1 by interacting with integrin-*α*7 in lung epithelial cells. In addition, CS exposure suppressed the transport of alpha-1 antitrypsin, which is involved in protecting the lung from degradation, inflammation, and apoptosis [41]. In addition, exosomes also played a role in COPD exacerbation. Exosomes with CD144, CD31, and CD62E were notably enriched in patients during COPD exacerbation than in stable patients [42].

Thus, EV and exosome shedding promoted by the exposure of CS appears to contribute to the development of CS-related COPD. Understanding how EVs and exosomes contribute in this process and changes in the EVs and exosome contents, which may be used as biomarkers during disease progression, in response to cigarette smoking might help us to inquire into pathogenesis and develop novel therapeutic strategies.

### 4.3. Lung Cancer

EVs have been reported to be genetic cargo in lung cancer, which could be used for diagnostic, prognostic, and predictive biomarkers [43, 44]. Tumor-derived exosomal miR-1247-3p induces cancer-associated fibroblast activation to foster lung metastasis of liver cancer [45, 46]. Knockdown of TGF-*β*1 expression in human umbilical cord mesenchymal stem cells reverts their exosome-mediated EMT-promoting effect on lung cancer cells [47]. Exosomal contents were reported to be involved in anticancer drug resistance [48]. In addition, exosomes are involved in the crosstalk between lung cancer stem cells (CSC) and cancer-associated fibroblasts (CAF) [49], which is the primary component of the lung cancer microenvironment. CSCs interact with the CAFs via exosomes and could be activated by their crosstalk. In tumor-associated cells, alpha-smooth muscle actin (*α*-SMA) was identified as a specific marker for myofibroblasts in lung cancer [50].

However, few studies reported the involvement of EV in CS-induced lung cancer disease. EVs released from airway epithelial cells show pathological properties when exposed to extracts obtained from CS. As CS extract causes oxidative stress, the oxidative components were proved to be responsible for inducing EV release and subsequent progress to lung cancer [21]. Lung cancer is more common in people with HIV than in the general population [51], and a major risk factor in this is smoking. A role of EV in CS-mediated HIV-1 pathogenesis has been reported. CS is known to exacerbate HIV-1 pathogenesis, especially in monocytes, through the oxidative stress pathway. EVs are known to alter HIV-1 pathogenesis through intercellular communication. The key factors were suggested by a potential role of antioxidant enzymes, which are differentially packaged into CSC-exposed HIV-1-infected cell-derived EVs, on HIV-1 replication of recipient cells [46].

In addition, there are reports on bronchial epithelial cell-derived EVs modulated by CS, which revealed a crucial pathogenic role for CS in the development of lung tumors. Six members of the let-7 family, namely, let-7a, let-7b, let-7c, let-7f, let-7g, and let-7i, were reported to be significantly reduced in human bronchial epithelial cell-derived EVs, after exposure to CS extract [10, 52]. The expression levels of the let-7 family members are lower in lung cancer tissues compared with normal lung tissues. Alterations in these tumor-suppressive miRNAs contribute to lung tumor development, suggesting a causal link between the reduction in the expression of the let-7 family in EVs and lung carcinogenesis in response to CS exposure, via EV transfer-mediated communication among these pathological cells [48, 53]. Understanding how exosomes contribute in this process and the alterations in EV contents derived from CS-induced lung tumors during disease progression may help us to understand the mechanisms and develop novel treatment strategies.

### 4.4. Oral Cancer

Oral cancer, the sixth most common cancer worldwide, is a subtype of the head and neck cancer; most of these occurred in less developed regions. Oral squamous cell carcinoma (OSCC) constitutes over 90% of all cancers of the oral cavity [54, 55]. Cigarette smoking leads to oral cancer because of the exposure of oral epithelial cells to free radicals, reactive oxygen species, and reactive nitrogen species that contribute to oxidative damages [56]. In addition, CS caused a profound effect on salivary TNF-*α* and MMP-8, which are markers of periodontal disease, in chronic periodontitis subjects in comparison to healthy controls [57]. As cigarette smoking is considered the most prevalent risk factor for oral cancer, saliva analysis serves as a crucial diagnostic tool. Seven mRNA biomarkers (DUSP1, H3F3A, IL-1B, IL-8, OAZ1, S100P, and SAT) in OSCC saliva [58] and two miRNAs (miR-125a and miR-200a) in saliva were characterized as potential biomarkers for oral cancer detection [59].

Recently, the field of salivary exosomics, which focuses on the nucleic acids and proteins in salivary exosomes as potential cancer biomarkers, has been rapidly expanding [60–62]. The use of salivary exosomes is promising due to their ready availability and noninvasiveness. Therefore, oral cancer-derived exosomes in saliva have great potential as biomarkers for cancer diagnosis. The exosomal miRNAs, miR-342-3p and miR-1246, were elevated in exosomes from oral cancer. Exosomal miRNA transfer from highly metastatic oral cancer cells to cells with low malignancy resulted in rapid cell proliferation, compared to the non-exosome-applied control cells by Sakha et al. [63].

However, the information on the saliva exosome research, targeting oral cancer in response to CS, is not available. Moreover, CS-induced salivary exosome shedding in oral cancers has not been reported yet. It is necessary to expand research on salivary exosomes, integrating current and past data and linking salivary exosomics with other biomedical research.

## 5. EVs as Potential Biomarkers in Exposure to CS for Chronic Diseases

Recently, diverse EVs and exosomal biomarkers have been reported to be potential diagnostic biomarkers for several diseases. However, CS-induced EV and exosomal biomarkers, miRNAs, and proteins, for CS-related diseases including CVD, COPD, and cancers, are limited.  Table 1 summarizes the current data on potential EVs and exosomal biomarkers and their corresponding diseases.

## 6. Conclusions and Perspective

Chronic exposure to cigarette smoke is the major risk factor for numerous diseases. Several studies cited the gaps between acute *in vitro* CS exposure and chronic *in vivo* CS exposure in smokers [12, 17]. Therefore, development of a suitable *in vitro* exposure model that replicates/mimics *in vivo* exposure is required for intensive research to fully elucidate the functional properties of CS-induced EV shedding, which is implicated in disease pathogenesis.

It is generally accepted that chronic exposure to environmental toxicants, including CS, could stimulate EVs and exosome biogenesis and the subsequent release that is associated with disease onset. However, how CS exposure triggers intracellular signal transductions that affect EVs and exosomes and how the subsequent EV and exosome release modulates a series of cellular events responsible for disease development remain unclear so far. Therefore, further studies are needed for elucidating the network between EVs and exosome alteration, which is implicated in CS exposure and disease progression. A wide range of EV and exosome studies depicting the diagnostic or therapeutic feasibility of them are in clinical test [67, 68]. However, it is premature to find the links between EV and exosome alterations and CS-specific diseases. At present, *in vivo*, preclinical, and clinical data do not significantly correlate with the *in vitro* data. Moreover, development of exosome isolation technology for obtaining pure and abundant exosomes is needed to overcome limitations in integrating and validating current findings. Finally, functional studies of CS-induced EVs and exosomes open up promising research leading to the development of diagnostic and therapeutic EVs and exosomes in CS-associated diseases.

## Figures and Tables

**Table 1:** Potential biomarkers of EVs and exosomes in response to cigarette smoke in chronic diseases.

Components	Species	Sources	Vesicle	Disease	Reference
miRNA					
miR-378a, miR-379, miR-139-5p, miR-200b-5p	Human	Plasma	Exosome	Lung cancer	[64]
miR-7, miR-20a, miR-28-3p, miR-34c-5p, miR-100	Human	Serum	Exosome	COPD	[65]
let-7d, miR-191 miR-125a, miR-126	Human Mouse	Cell supernatant Plasma	Endothelial microparticles (EMPs)	COPD	[8]
let-7e, let-7g, miR-26b	Human	Bronchoalveolar lavage (BAL)	EVs	COPD Lung cancer	[14]
miR-210	Human	Cell supernatant	EVs	COPD	[10]
miR-223, miR-29b	Human	Plasma	Microvesicle	CVD	[31]
Protein					
LRG1	Human	Urine	Exosome	NSCLC	[66]
*μ*-Calpain, m-calpain	Human	Cell supernatant	Vesicles	Lung cancer	[11]
